# Pig-hunting dogs are an at-risk population for canine heartworm (*Dirofilaria immitis*) infection in eastern Australia

**DOI:** 10.1186/s13071-020-3943-4

**Published:** 2020-02-13

**Authors:** Bronwyn Orr, Gemma Ma, Wei Ling Koh, Richard Malik, Jacqui M. Norris, Mark E. Westman, Denise Wigney, Graeme Brown, Michael P. Ward, Jan Šlapeta

**Affiliations:** 1 Sydney School of Veterinary Science, The University of Sydney, Sydney, NSW 2006 Australia; 2 Centre for Veterinary Education, The University of Sydney, Sydney, NSW 2006 Australia; 3 Greyhound Adoption Program (NSW), PO Box 24, Belrose West, NSW 2085 Australia

**Keywords:** Heartworm, *Dirofilaria immitis*, Canine, Pig-hunting, Australia, Prevalence, At-risk population

## Abstract

**Background:**

Canine heartworm disease, caused by *Dirofilaria immitis*, has global veterinary importance. In Australia, the prevalence of canine heartworm infection decreased markedly following the introduction of over-the-counter macrocyclic lactones. We aimed to estimate the prevalence of canine heartworm infection in at-risk populations of dogs in eastern Australia and analyse published prevalence data from Australia.

**Methods:**

In total, 566 dogs from eastern Australia were tested for the presence of *D. immitis* antigen. Four cohorts were studied: pig-hunting dogs from Queensland (Cohort 1, *n* = 104), dogs from remote New South Wales (NSW) (Cohort 2, *n* = 332), urban pets from rural NSW (Cohort 3, *n* = 45) and ex-racing Greyhounds from Sydney, NSW (Cohort 4, *n* = 85). Serum samples were screened for *D. immitis* antigen using a reference laboratory microwell-based assay (DiroChek^®^) or a point-of-care immunochromatography test kit (Anigen Rapid^®^). Risk factors associated with the odds of *D. immitis* antigen seropositivity were identified using binary logistic regression models. Seropositive blood samples were tested for the presence and quantity of *D. immitis* DNA using a species specific real-time (q)PCR assay. A metanalysis of the Australian canine heartworm literature was conducted.

**Results:**

The prevalence of dirofilariasis in pig-hunting dogs from Queensland (Cohort 1) was 12.5% (95% CI: 6.5–18.9%), with a subpopulation of dogs from Central Queensland having a prevalence of 21% (95% CI: 12.3–33.4%). Age was significantly associated with *D. immitis* antigen seropositivity (increased risk with increased age). The odds of being > 5 years *versus* ≤ 5 years was 3.7-times (95% CI: 1.1–12.5) greater in antigen positive versus antigen negative dogs. No *D. immitis* antigen positive dogs were detected in dogs from NSW (Cohorts 2–4). The Australian canine heartworm disease literature includes 98 peer-reviewed publications (1901–2019) with 30 studies reporting on *D. immitis* prevalence in dogs. Throughout the publication peak period (1980s), the primary antemortem diagnostic test was detection of microfilariae.

**Conclusions:**

Canine heartworm infection in dogs used for pig hunting is a previously unexplored topic in Australia. Pig-hunting dogs are infected with canine heartworm in Queensland, Australia, placing pet dogs and cats at increased risk of infection.
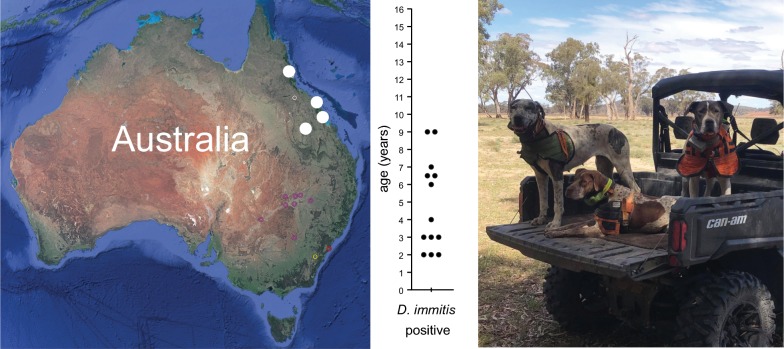

## Background

Canine heartworm disease (dirofilariasis) is caused by the filarial nematode *Dirofilaria immitis* [1, 2]. Left untreated, canine heartworm disease can become life-threatening, with the parasite’s tropism for the pulmonary arterial vasculature causing inflammation, thrombo-embolism, pulmonary hypertension and eventually, right-sided heart failure [3, 4].

Historically, Australia experienced high rates of canine heartworm disease across several regions in the 1970s and 1980s. During this era, the prevalence of canine heartworm in Queensland, Northern Territory and New South Wales (NSW) was reported to range from 30% to 100%, with the tendency for higher prevalence to occur in the tropical and subtropical regions of northern Australia [5–10]. Since then, the prevalence of canine heartworm in Australia has decreased progressively. By the early 1990s, a wide range of once-a-month oral or ‘spot-on’ macrocyclic lactones (MLs) for canine heartworm prevention had become available in Australia. These preventatives did not require a prescription and could be purchased across the counter from veterinary clinics, pet stores and (later) from online retail outlets and supermarkets [11, 12]. In 2016, despite these measures, an apparent re-emergence of *D. immitis* infection and canine heartworm disease was documented in pet dogs in Central Queensland [13]. Apart from the impact of prophylactic therapy, factors affecting the prevalence of canine heartworm disease in Australia are incompletely understood.

**Fig. 1:**
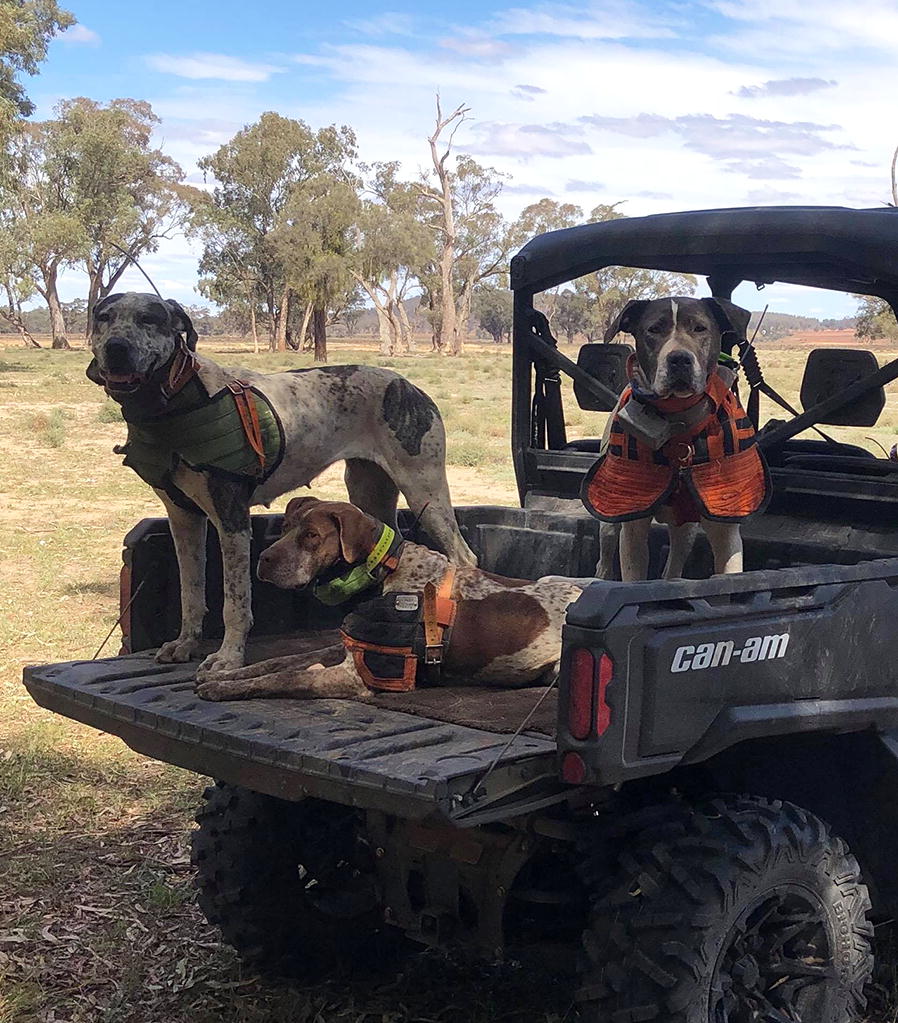
Pig-hunting dogs in Australia. Image courtesy of John Calvani

For *D. immitis* to successfully (re)emerge and sustain its life-cycle, certain pre-requisites need to be met. First, the parasite is mosquito-borne. In Australia, the competent vectors have been shown to be mosquitoes of the genera *Aedes*, *Culex* and *Anopheles* [10, 14, 15]. Secondly, there needs to be an absence of prophylactic treatment with MLs, either through poor or absent client compliance or inadequate dosing, as this probably represents the most common reason for canine heartworm infection globally [16–18]. Finally, there needs to be competent species acting as reservoirs for *D. immitis* such as wild dogs, dingoes and owned dogs not receiving prophylaxis [19]. Identifying and monitoring canine cohorts that do not receive regular heartworm prevention (at-risk populations) is imperative, particularly in regions with low endemicity. One possible group in Australia is pig-hunting dogs. Hunting feral pigs (known as hogs in the USA) is a popular activity in Australia, with an estimated 156,000 dogs kept specifically for this purpose [20] (Fig. 1). Recent enquiries into the health and welfare of pig-hunting dogs in Australia and New Zealand suggested they were at high risk of exposure to numerous infectious diseases and received minimal veterinary preventive care [20, 21].

In this study, we aimed to identify at-risk populations of dogs based on a history of incomplete canine heartworm (*D. immitis*) prevention. To do so, we used *D. immitis* antigen testing and positive samples were tested for the presence of *D. immitis* DNA. Four populations of dogs with moderate or no canine heartworm prevention were tested, including pig-hunting dogs, remote community dogs, owned urban dogs and ex-racing Greyhounds. For the cohort of pig-hunting dogs, reference laboratory *D. immitis* antigen testing was performed to evaluate the accuracy of a point-of-care immunomigration *D. immitis* antigen test. The context of canine heartworm in Australia is explained using a historical review (metanalysis) of published data.

## Methods

### Dog cohorts from Queensland and New South Wales, Australia

**Fig. 2:**
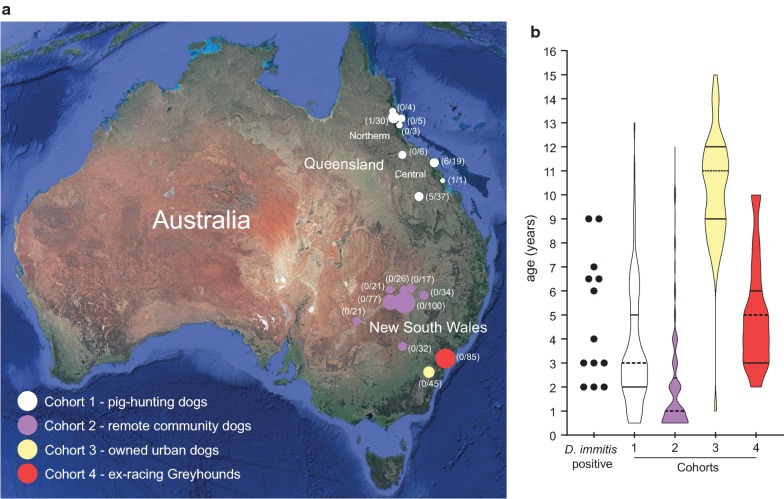
Dogs surveyed for the presence of *Dirofilaria immitis* antigen in Australia. **a** Geographical distribution of dogs tested for heartworm as part of 2016–2019 prevalence surveys. The size of the circle at each location in New South Wales and Queensland (Central and Northern) is proportional to the number of tested dogs, number in brackets indicates number of *D. immitis* antigen positive dogs / total number of tested dogs. **b** Violin plots of distribution of age of 566 dogs sampled as part of four distinct cohorts, mean and quartiles are show within the violins. On the far left of the figure is a scatter dot plot of the individual ages of 13 antigen-positive dogs

Four canine cohorts in Australia were sampled to determine the presence of canine heartworm (*D. immitis*) antigen (Fig. 2, Table 1). In total, 566 dogs were sampled. Whole blood samples were separated into serum and stored at − 20 °C prior to testing at the Sydney School of Veterinary Science (SSVS), University of Sydney.

**Table 1:** Summary of 566 dogs tested for *Dirofilaria immitis* antigen across eastern Australia 2016–2019

Cohort	Locality	Age^a^	Positive/total		Canine heartworm prophylaxis
			DiroChek^®^	Anigen Rapid^®^	
Queensland					
Cohort 1	Northern	3 (0.1–13)	1^b^/42	1^b^/42	None or intermittent
	Central	3 (0.1–9)	12/62	11/62	None or intermittent
New South Wales					
Cohort 2	Remote western	1 (0.5–12)	0/332	nd	None
Cohort 3	Goulburn	9 (1–15)	0/45	nd	None
Cohort 4	Greater Sydney	5 (2–9)	nd	0/85	Rigorous in 54% (46/85)

^a^Median (minimum-maximum), in years

^b^A 9-year-old male relocated from Central Queensland

*Abbreviations*: nd, not determined; DiroChek^®^, DiroChek^®^ Heartworm Antigen Test Kit (Zoetis, NJ); Anigen Rapid^®^, Rapid Test Kit Heartworm (CHW) Ag2.0 (Anigen, BioNote Inc., Seoul, South Korea)

Cohort 1 [Pig-hunting dogs (*n* = 104)] were sampled from August to November 2018 from towns across Central and North Queensland, Australia. Locations included Sarina, Clermont, Proserpine, Charters Towers, Innisfail, Tully, Malanda and Atherton. Dogs sampled had been used for hunting feral pigs (hogs), and generally were large, mixed breed dogs living outdoors in kennels or yards. Based on currently unpublished survey data from the owners, only intermittent canine heartworm prophylaxis was used, if at all.

Cohort 2 [Remote community dogs (*n* = 332)], predominantly crossbreeds, were sampled from six communities across remote and very remote western New South Wales between 2016–2018. Dogs were recruited from the participants of Royal Society for the Prevention of Cruelty to Animals (RSPCA) NSW Community Companion Animal Health Programs, a service provided for people on low incomes, many of whom identify as Aboriginal. These dogs have limited or no access to veterinary treatment including canine heartworm prophylaxis.

Cohort 3 [Owned urban dogs (*n* = 45)] were sampled between June and September 2018 in Goulburn, a small inland city in NSW, Australia. These dogs were all pets visiting a local veterinary clinic, none of which were receiving heartworm prophylaxis from their owners at the time of sampling.

Cohort 4 [Adopted ex-racing Greyhounds (*n* = 85)] were sampled between January 2018 and November 2019 in the Greater Sydney Region, NSW. These dogs were all part of Greyhound Adoption Program (NSW) Inc, having retired from racing and currently living as pet dogs. More than half of these dogs (46/85, 54%; self-reported by owners) were receiving monthly heartworm preventatives, although their treatment history was not known in any more detail.

### Testing for *Dirofilaria immitis* antigen

Serum samples from Cohorts 1–3 were tested using DiroChek^®^ Heartworm Antigen Test Kits (Zoetis, Parsippany, USA), a qualitative microwell-based enzyme-linked immunosorbent assay (ELISA) for the detection of adult female *D. immitis* antigen. DiroChek^®^ is intended for use by diagnostic laboratories and was used according to manufacturer’s instructions; 5 min after the addition of the final solution, the 96-well plate or 8-well strip with manufacturers positive and negative controls were visually examined for a clear to blue color change. All blue colored wells, regardless of intensity, were considered antigen positive or, if no blue color was detected, samples were considered to have ‘no detectable antigen’ (NDA).

Serum samples from Cohorts 1 and 4 were tested using point-of-care Anigen Rapid^®^ Canine Heartworm (CHW) Ag 2.0 Test Kits (BioNote, Gyeonggi-do, Korea) a chromatographic immunoassay for the detection of *D. immitis* antigen. Sensitivity and specificity of Anigen Rapid^®^ CHW Ag 2.0 for *D. immitis* antigen have been reported as 99.5% and 94%, respectively [22]. The Anigen Rapid^®^ kit used in the study by Henry et al. [22] is identical to the one marketed and sold in Australia (Mark Thacker, Life Bioscience, Oakleigh, Australia, personal communication).

Knott’s test for the identification of *D. immitis* microfilariae (Mff) was not performed due to the logistical constrains, remote sampling locations and lack of laboratory resources in the field.

### *Dirofilaria immitis* specific real-time PCR

Blood clot samples (200 μl) from *D. immitis* antigen positive dogs and a random selection of NDA *D. immitis* dogs (Cohort 1) were used for molecular studies. Genomic DNA was isolated using PowerMag^®^ Blood DNA/RNA Isolation Kits (Qiagen, Chadstone, Australia) optimised for the KingFisher^®^ Duo Prime Purification System (Thermo-Fisher Scientific, Scoresby, Australia). DNA isolation was performed as a batch of 11 blood samples and an extraction blank. DNA eluted in 100 μl was stored in aliquots at − 20 °C prior to analysis.

Presence of canine DNA was verified using real-time PCR (qPCR) to amplify partial canine glyceraldehyde-3-phosphate dehydrogenase (GAPDH), forward primer (S0631): 5ʹ-TCA ACG GAT TTG GCC GTA TTG G-3ʹ and reverse primer (S0634): 5ʹ-TGA AGG GGT CAT TGA TGG CG-3ʹ with probe (S0632): 5ʹ-HEX-CAG GGC TGC TTT TAA CTC TGG CAA AGT GGA-BHQ1-3ʹ; RTPrimerDB ID: 1193 [23]. PCR cycling conditions included an initial step at 95 °C for 3 min followed by 40 two-step cycles of 95 °C for 5 s and 60 °C for 15 s. The final qPCR reaction mixture (20 μl) included 10 μl of *Sso*Advanced Universal Probes Supermix (BioRad, Gladesville, Australia), each primer at 400 nM concentrations, probe at 100 nM concentration, PCR-grade water, and 2 μl of DNA template. PCR testing was performed using a CFX96 Touch™ Real-Time PCR Detection System with the corresponding CFX Manager v.3.1 software (BioRad, Gladesville, Australia). The arbitrary qPCR threshold was determined automatically using default settings and threshold cycle (C_q_)-values reported.

A species-specific *D. immitis* qPCR assay and microfilariae (Mff) quantification was performed, as described [13]. Primers targeting the *cox*1 gene fragment (~ 203 bp) of *D. immitis* were used, consisting of forward primer (S0582): 5ʹ-AGT GTA GAG GGT CAG CCT GAG TTA-3ʹ and reverse primer (S0583): 5ʹ-ACA GGC ACT GAC AAT ACC AAT-3ʹ) [24]. The final qPCR reaction mixture (20 μl) included 10 μl of *Sso*Advanced Universal SYBR^®^ Green Supermix (BioRad, Gladesville, Australia), each primer at 400 nM concentrations, PCR-grade water, and 2 μl of DNA template. Each sample was run in duplicate with 10-fold serial dilutions (1.96 × 10^2^ to 1.96 × 10^7^ copies per reaction) of a known concentration of plasmid DNA containing the *D. immitis cox*1 gene synthetised by GeneArt (Thermo Fisher Scientific, Scoresby, Australia) and with negative (no template) controls, as described [13]. Amplification was performed as follows: initial denaturing step at 95 °C for 3 min, followed by 40 cycles of denaturing (5 s at 95 °C) and annealing (15 s at 62 °C). A final melting curve was produced by heating the product from 60 °C to 90 °C for 5 s at 0.5 °C increments. PCR testing was performed using a CFX95 Touch™ Real-Time PCR Detection System (BioRad Laboratories, Gladesville, Australia). C_q_ values and standard curves were determined CFX Manager ™ Software v. 3.1 (BioRad Laboratories, Gladesville, Australia). Any sample that amplified with a C_q_-value < 40 was sent for DNA purification and sequencing using amplification primers (Macrogen Inc., Seoul, Korea). Sequence chromatographs were manually inspected and compared to a reference *D. immitis cox*1 sequence (NC_005305) using CLC Main Workbench 6.9.1. (CLC Bio, Qiagen, Chadstone, Australia).

### Review of published canine heartworm prevalence data in Australia

Using the University of Sydney’s *Library Search* engine, in August 2019 we searched the peer-reviewed literature for the following terms: ‘heartworm AND Australia’ (789 results), ‘*Dirofilaria immitis* AND Australia’ (793 results) and ‘dirofilariasis AND Australia’ (275 results), with no restriction on material type and date and the search terms located in ‘any field’ within each database entry. The *Library Search* engine searches across library resources, including the library catalogue and journal databases such as Medline, Web of Science and Scopus. In addition, we conducted a second search in the *Australian Veterinary Journal* archives using the following terms: ‘heartworm’, ‘*Dirofilaria immitis’* and ‘dirofilariasis’. We examined peer-reviewed references from a specific PhD thesis (Martin TE, 1986: ‘Prevalence and diagnosis of infection with *Dirofilaria immitis* in dogs’, University of Sydney) as well as reference lists from all reviewed articles. Once collated, retrieved articles were manually inspected to confirm the presence of heartworm prevalence data and then reviewed in more detail. Inclusion criteria for retained references included primary data on *D. immitis* prevalence in dogs, regardless of the method used. For each retained reference, information on the year of study, study area, sample size, number of samples positive for *D. immitis* and test used was analysed.

### Statistical analysis

Confidence intervals (95% CI) were calculated using the modified Wald method. For Cohort 1, the association between heartworm antigen status (positive, negative) and age (≤ 5 years *versus* > 5 years), type (‘Bull Arab’ or ‘Bull Arab crossbreed’ *versus* others), sex (male *versus* female) and location (inland *versus* coastal) was tested using binary logistic regression models (*P* < 0.05). Possible associations were assessed using odds ratios (IBM^®^ SPSS Statistics v24). Non-parametric Kruskal-Wallis one-way ANOVA testing with Dunn’s multiple *post hoc* comparisons was used for age of cohorts because of the non-normal age distribution within each cohort (GraphPad Prism 8.2.1). The sensitivity and specificity of Anigen Rapid^®^ were calculated using results from Cohort 1 and the DiroChek^®^ result as the gold standard, and the degree of agreement between Anigen Rapid^®^ and DiroChek^®^ quantified by determining the Kappa statistic (GraphPad Prism 8.2.1).

## Results

Of the 566 dogs tested for *D. immitis* antigen, 13/566 (2.3%; 95% CI: 1.3–3.9%) were positive (Table 1, Fig. 2a). All 13 antigen positive results were from pig-hunting dogs in Queensland (Cohort 1), comprised of nine males and four females (13/104; 13%; 95% CI: 6.5–18.9%). All but one *D. immitis* antigen positive dogs came from Sarina, Proserpine or Clermont (the Mackay and Whitsundays region of Central Queensland). Considering this region alone, 12/57 dogs tested antigen positive (21%, 95% CI: 12.3–33.4%). The remaining *D. immitis* antigen positive dog came from Malanda (Atherton Tablelands region of Far North Queensland), although this dog had been imported from Central Queensland.

No dogs tested in NSW (Cohorts 2–4, *n* = 462) returned a positive antigen test result and all were therefore considered NDA. The age distribution of dogs was significantly different between the four cohorts (Kruskal-Wallis one-way ANOVA, *H* = 237.1, *df* = 3, *P* < 0.0001), with all cohorts being significantly different from each other (Dunn’s multiple comparisons, *P* < 0.05). The median age of Cohort 2 was the lowest, followed by Cohorts 3, 1 and 4 (Fig. 2b, Table 1).

In Cohort 1, antigen status was significantly (*P* = 0.03) associated with age: the odds of being > 5 years *versus* ≤ 5 years was 3.73-times (95% CI: 1.1–12.5) greater in seropositive *versus* seronegative dogs (binary logistic regression models). Seropositivity was not associated with sex (*P* = 0.37; male *versus* female) or type (*P* = 0.6; Bull Arab and crossbreds *versus* others). Location (*n* = 8) as a fixed effect was not significantly (*P* = 0.54) associated with antigen status. However, location categorised as coastal *versus* inland was significantly (*P* = 0.03) associated with serostatus: the odds of being coastal *versus* inland was 3.89-times (95% CI: 1.2–12.8) higher in antigen positive *versus* antigen negative dogs. Finally, both age and location were significantly associated with serostatus when included in the same logistic regression model (Table 2). This model adequately fitted the data (Hosmer & Lemeshow test, *χ*^2^ = 1082, *df* = 2, *P* = 0.58), while the interaction between age and location was not significant (*P* = 0.30).

**Table 2:** Risk factors associated with the odds of *Dirofilaria immitis* antigen seropositivity of 104 pig-hunting dogs in North Queensland, Australia

Variable	Category	Beta	SE	*P-*value	Odds ratio	95% CI
Age	≤ 5 years	0	–	–	1	–
	> 5 years	1.331	0.642	0.038	3.786	1.076–13.326
Location	Inland	0	–	–	1	–
	Coastal	1.371	0.629	0.029	3.940	1.148–13.522

*Abbreviations*: SE, standard error; 95% CI, 95% confidence interval

The sensitivity of the Anigen Rapid^®^ test was 92% (95% CI: 62.1–99.6%) and specificity was 100% (95% CI: 95.1–100%), while the kappa statistic was 0.96 (95% CI: 0.87–1.00) (Table 3).

**Table 3:** Summary of molecular confirmation of *Dirofilaria immitis* using species specific qPCR

Sample ID	DiroChek^®a^	Anigen Rapid^®b^	Estimated *D. immitis* (Mff.ml^−1^)	Host (dog) C_q_(GAPDH)
BO#231	Positive	Positive	< 5	26.17
BO#103	Positive	Positive	< 5	24.54
BO#135	Positive	NDA	503	23.76
BO#102	Positive	Positive	537	25.38
BO#55	Positive	Positive	6591	28.13
BO#56	Positive	Positive	7665	27.13
BO#138	Positive	Positive	22,050	24.34
BO#134	Positive	Positive	0	23.81
BO#136	Positive	Positive	0	24.71
BO#168	Positive	Positive	0	26.61
BO#58	Positive	Positive	0	27.23

^a^DiroChek^®^ Heartworm Antigen Test Kit (Zoetis, NJ)

^b^Anigen Rapid^®^ Test Kit Heartworm (CHW) Ag2.0 (Anigen, BioNote Inc., Seoul, South Korea)

*Abbreviations*: NDA, no detectable antigen; Mff, *D. immitis* microfilariae

To determine if the *D. immitis* antigen positive pig-hunting dogs were a source of *D. immitis* for mosquitoes (i.e. that they were microfilaraemic), we used available blood clots from 11/13 antigen positive dogs and a selection (*n* = 9) of antigen-negative dogs (Table 3). DNA was successfully purified and canine GAPDH amplified (C_q_ values: 21.8–31.5) from 20 canine blood samples. Blank DNA samples remained negative in the canine GAPDH assay. Seven Cohort 1 samples (7/11, 64%) from *D. immitis* antigen positive dogs were considered positive for *D. immitis* microfilariae DNA (Table 3). The calculated microfilariae concentration ranged from < 1 to 22,050 microfilariae/ml (Table 3). Four of 11 samples returned negative results (0 to < 5) for *D. immitis* DNA for samples that were *D. immitis* antigen positive (Table 3). These negative results are consistent with a concentration of microfilariae < 5/ml of blood, because we used 200 µl of blood for DNA isolation and thus required at least 1 microfilaria for a positive DNA result. All antigen-negative samples (*n* = 9) yielded negative results for the presence of *D. immitis* microfilariae DNA (Table 3), as expected. For the *D. immitis* DNA positive samples, DNA sequencing of the *cox*1 PCR amplicon was 100% identical with the *D. immitis cox*1 gene reference sequence (GenBank: NC_005305).

**Fig. 3:**
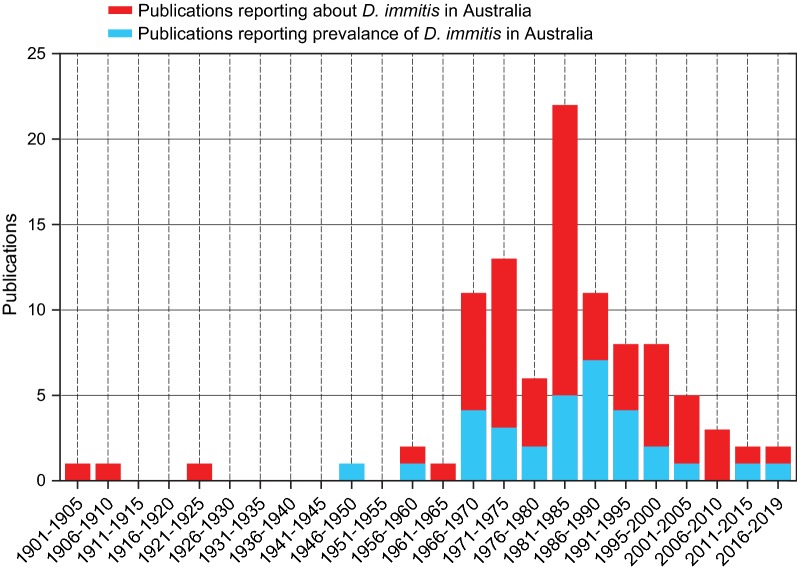
Histogram of published studies on *Dirofilaria immitis* in Australia from 1901 to 2019. All articles were manually inspected to confirm the presence *D. immitis* data in Australia (red, *n*** = **98) and studies reporting prevalence data of *D. immitis* were identified (blue, *n*** = **32). Inclusion criteria for retained references included primary data on *D. immitis* prevalence in dogs, regardless of the method used

Searches to determine the historic prevalence of canine heartworm disease in Australia yielded 98 peer-reviewed publications spanning the years 1901–2019 (Figure 3). After applying our inclusion criteria, 32 studies remained (Figure 3, Table 4). Of these, 30 studies reported on *D. immitis* prevalence in dogs, and two studies included data on *D. immitis* prevalence in red foxes (Table 4). Most publications reporting *D. immitis* in canids were published during 1966–2005, with a peak of 12 publications during the 1980s (Figure 3). Throughout this peak period, the primary antemortem diagnostic test was detection of microfilariae (Table 4).

**Table 4:** Summary of canine heartworm prevalence data in Australia from published studies between 1901–2019

Location	Year(s)	Sample size	Sample source	Method of detection	Prevalence (%)	Reference
Queensland						
Brisbane	1959	Unknown	Dogs	Necropsy	10 (unspecified)	[37]
Brisbane	1964–1965	761	RSPCA dogs	Necropsy and/or Mff	24 (183/761; 95% CI: 21.1–27.2)	[6]
Brisbane	1968	114	Pound dogs	Unknown	17 (unspecified)	[38]
Townsville	1969	94	Vet clinic dogs	Mff	37 (35/94; 95% CI: 28.1–47.3)	[6]
Brisbane		296			21 (61/296; 95% CI: 16.4–25.6)	
Brisbane	1969–1971	238	Dogs	Mff	15 (37/238; 95% CI: 11.5–20.7)	[10]
Cherbourg		16	Dogs		6 (1/16; 95% CI: < 0.1–30.3)	
Edward River		36	Dogs		85 (31/36; 95% CI: 70.9–94.4)	
Aurukun		23	Dogs		90 (20/23; 95% CI: 67.0–96.3)	
Bamaga		48	Dogs		25 (12/48; 95% CI: 14.8–38.9)	
Hope Vale		86	Dogs		0 (0/86; 95% CI: 0–5.1)	
Mornington Island		14	Dogs		0 (0/14; 95% CI: 0–25.2)	
Kowanyama		84	Dogs	Necropsy	88 (78/84; 95% CI: 85.0–97.0)	[10]
Townsville	1972	28	Owned dogs	Mff	68 (unspecified)	[39]
Brisbane	1972–1976	480	Dogs	Mff	36 (171/480; 95% CI: 31.5–40.0)	[10]
Cherbourg		80	Dogs		8 (6/80; 95% CI: 3.2–15.7)	
Kowanyama		45	Dogs		64 (29/45; 95% CI: 49.8–76.8)	
Edward River		23	Dogs		78 (18/23; 95% CI: 57.7–90.8)	
Bamaga		45	Dogs		71 (32/45; 95% CI: 56.5–82.4)	
Brisbane	1979	120	Pound dogs	Mff	36 (43/120; 95% CI: 27.8–44.7)	[7]
Brisbane	1981	100	Stray puppies (> 8 weeks)	Mff	1 (1/100; 95% CI: < 0.1–6.0)	[40]
Brisbane	1986	57	Pound dogs	Necropsy	60 (34/57; 95% CI: 46.7–71.4)	[41]
Brisbane	1986	59	Pound dogs	Necropsy	64 (35/59; 95% CI: 46.6–70.9)	[42]
Brisbane	1988	125	Stray dogs	Necropsy^a^	42 (52/125; 95% CI: 33. 3–50.7)	[28]
Brisbane	1989	100	Dogs	Necropsy	22 (22/100; 95% CI: 14.9–31.1)	[12]
Brisbane	1991	272	Pound dogs	Necropsy	49 (134/272; 95% CI: 43.4–55.2)	[25]
Brisbane	1993	57	Pound dogs	Necropsy	60 (34/57; 95% CI: 46.7–71.4)	[43]
Townsville	2001	Unknown	Pound dogs	Unknown	15 (unspecified)	[44]
	2002	27	Wild dogs		56 (15/27; 95% CI: 37.3–72.4)	
Yarrabah	2008–2012	51	Remote community dogs	SNAP (Idexx)	2 (1/51; 95% CI: < 0.1–11.3)	[45]
Cairns	2007–2013	23	Wild + urban fringe dingoes	Necropsy, Mff, SNAP (Idexx)	44 (10/23; 95% CI: 25.6–63.2)	[19]
Atherton	2007–2013	5	Urban fringe dingoes	Necropsy	0 (0/5; 95% CI: 0–48.9)	[19]
New South Wales						
Murrumbidgee Irrigation Area (Southern NSW)	1966	Unknown	Necropsy dogs	Necropsy	50 (unspecified)	[46]
Sydney	1968	62	Dogs	Mff	19 (12/62; 95% CI: 11.3–31.0)	[47]
Sydney	1971–1972	495	Pound dogs	Necropsy	4 (21/495; 95% CI: 2.8–6.4)	[26]
Sydney	1971	40	Vet clinic dogs	Mff	8 (3/40; 95% CI: 1.9–20.6)	[26]
Sydney	1972	339	Pound dogs	Necropsy	3 (9/339; 95% CI: 1.3–5.0)	[48]
Hunter Valley/Coastal NSW	1979–1981	331	Greyhounds	Mff	11 (36/331; 95% CI: 7.9–14.7)	[9]
Sydney	1981–1983	405	Vet clinic dogs	Mff	13 (51/405; 95% CI 9.7–16.2)	[49]
Sydney	1982–1983	68	Red foxes *Vulpes vulpes*	Necropsy	9 (6/68; 95% CI: 3. 8–18.3)	[49]
Sydney	1983	100	Pound dogs	Necropsy	50 (50/100; 95% CI: 40.4–59.6)	[50]
Sydney	1987	100	Pound dogs	Necropsy^a^	24 (24/100; 95% CI: 16.6–33.3)	[29]
Sydney	1987	100	Pound dogs	Necropsy	32 (32/100; 95%CI: 23.7 to 41.7)	[51]
Sydney	1992	304	Pound dogs	Mff^a^	12 (36/304; 95% CI: 8.7–16.0)	[5]
		100		Necropsy^a^	15 (15/100; 95% CI: 9.2–23.4)	
Collarenebri	2008–2009	39	Remote community dogs	SNAP (Idexx)	0 (0/39; 95% CI: 0–10.7)	[45]
Goodooga	2008–2009	19	Remote community dogs	SNAP (Idexx)	21 (4/19; 95% CI: 8.0–43.9)	[45]
Victoria						
Melbourne	1946	174	Ownerless dogs	Necropsy	0 (0/174; 95% CI: 0–2.6)	[52]
North-eastern Victoria	1978	752	Pound dogs (734)	Necropsy	2 (15/734; 95% CI: 1.2–3.4)	[53]
			Greyhounds (18)		28 (5/18; 95% CI: 12.2–51.2)	
Melbourne	1991–1994	125	Red foxes (*Vulpes vulpes*)	Dirochek^®^ (Symbiotics)	6 (8/125; 95% CI: 3.1–12.3)	[54]
Northern Territory						
Darwin	1963	Unknown	Dogs	Unknown	70 (unspecified)	[6]
Alice Springs	1972–1976	98	Vet clinic dogs	Mff	13 (12/98; 95% CI: 0.1–20.3)	[10]
Docker River + Indulkana		73	Remote community dogs	Mff	0 (0/73; 95% CI: 0–6.0)	[10]
Katherine	1988	32	Dingoes	Necropsy	56 (18/32; 95% CI: 39.3–71.9)	[55]
Ti Tree	2007–2009	48	Remote community dogs	SNAP (Idexx)	2.5 (4/48; 95% CI: 2.8–20.1)	[45]
Tiwi Islands	2008–2009	27	Remote community dogs	SNAP (Idexx)	0 (0/27; 95% CI: 0–14.8)	[45]
South Australia						
South Australia	1988–1991	1658	Dogs	Mff	1 (19/1658; 95% CI: 0.7–1.8)	[8]
Western Australia						
Kimberly	1993	58	Remote community dogs	Necropsy	0 (0/58; 95% CI: 0–7.4)	[56]
Bidyadanga	2008	15	Remote community dogs	SNAP (Idexx)	0 (0/15; 95% CI: 0–23.9)	[45]

^a^Validation of DiroChek^®^ Symbiotics Corp. USA supplied by Commonwealth Serum Laboratories, Parkville, Victoria (now owned by Zoetis, NJ)

*Abbreviations*: 95% CI, 95% confidence interval; Mff, detection of microfilariae

## Discussion

The only canine heartworm (*D. immitis*) antigen positive dogs detected in our survey of 566 dogs were pig-hunting dogs from Queensland. All 13 *D. immitis* antigen-positive dogs, including one domiciled in Far North Queensland at the time of testing, had spent time in the Mackay and Whitsundays regions of Central Queensland. Recent research by Nguyen et al. [13] demonstrated the presence of *D. immitis* in this region in dogs visiting veterinary practices. More than one-fifth of the pig-hunting dogs tested in Central Queensland were *D. immitis* antigen-positive. The current prevalence of *D. immitis* antigen seropositivity in pig-hunting dogs in this region is comparable to the historical *D. immitis* prevalence data in pound dogs based on necropsy or microfilaraemia in Queensland during 1960–1990 [6, 7, 10, 25]. A significantly higher prevalence of *D. immitis* antigen seropositivity in dogs older than five years of age compared to younger dogs has previously been reported in Australia, and is a consequence of increased likelihood of infection with the cumulative exposure to more mosquito bites throughout life [6, 26]. In a large Australian study [10], no dog younger than 9 months showed presence of *D. immitis* microfilariae, and male dogs were more often positive then females. Possibly due to smaller samples sizes of at-risk dogs in our study, there was no difference observed in the sex of the dog in relation to antigen seropositivity.

Pig-hunting dogs are thought to receive minimal veterinary and preventative health care and often travel widely with their owners during hunting expeditions [20, 21]. Pig-hunting dogs are generally robust, large breed, short-coated dogs that live outdoors [20], increasing their risk of being bitten by mosquitoes and thereby developing canine heartworm disease. Since they often reside close to pet dog populations, it will be important to investigate their potential as a reservoir for pet dogs sharing the same geography and receiving incomplete or lax canine heartworm preventative therapy.

In this study, we principally used the reference laboratory test, DiroChek^®^ Heartworm Antigen Test Kit, for detection of adult *D. immitis* antigen. Courtney et al. [27] reported DiroChek^®^ sensitivity to be 86% and specificity 97% in the USA. More recently, Henry et al. [22] re-evaluated the accuracy of DiroChek^®^ testing in the USA using a cohort of dogs with known *D. immitis* status, reporting test sensitivity of 99% (95% CI: 96.4–99.9%) and specificity of 96% (95% CI: 86.3–99.5%), including a cohort of 50 dogs with low worm burdens (1–5 female *D. immitis*; Mff in 58% of dogs). In Australia, the DiroChek^®^ has been tested extensively for its ability to detect *D. immitis* during the prepatent period and low heartworm burdens [5, 28, 29]. The DiroChek^®^ was validated in 1987 by testing 100 dogs from Sydney, NSW and immediately performing necropsy examination, with the test correctly identifying 15/24 infected dogs (sensitivity 62.5%) and 74/76 uninfected dogs (specificity 97.4%) [29]. Dogs returning a false negative result using DiroChek^®^ included four dogs with one male heartworm, three dogs with two male worms, one dog with one male and two female worms and one dog with five male worms [29]. In another group of 100 pound dogs from Sydney examined by necropsy, 8 dogs that were negative using DiroChek^®^ had adult heartworms at necropsy, four with prepatent infections and four with infections of three or less worms, all of one sex [5]. Similar results were obtained in a study that validated DiroChek^®^ in Brisbane, Queensland using a cohort of 125 necropsied dogs (52/125, 42% infected with *D. immitis*) with a reported sensitivity and specificity of 73.1% and 95.9%, respectively [28].

Henry et al. [22] demonstrated Mff absence in 19% of the necropsy positive dogs with female *D. immitis*, nevertheless absence of Mff was reported in dogs even with burden of > 40 female *D. immitis* (18%, 4/50). In Australia, absence of Mff was reported in 65% (15/26) and 20% (3/15) of *D. immitis* necropsy positive dogs from Brisbane [28] and Sydney [5], respectively. In our study, we applied species-specific PCR to detect the presence of microfilarial DNA, which was absent in four of DiroChek^®^ antigen positive dogs. These findings confirm that detection of microfilariae by filtration (Difil) or concentration (Knotts) techniques are complimentary to DiroChek^®^ to identify microfilariae-positive dogs prior to antigen testing and should be performed whenever screening at-risk dogs for *D. immitis*.

Detection of low burdens of *D. immitis* in dogs is difficult, because DiroChek^®^ sensitivity can be variable (53% in [28]; 62.5% in [29]; 86% in [27]) due to unknown worm burden distribution in the dog population. In areas of recent heartworm introduction, newly infected dogs will have no Mff due to the prepatent period of at least six months, single sex infections or burdens with less than two female heartworms. Additionally, the formation of immune complexes can further complicate diagnosis and detection of *D. immitis* antigen [30, 31].

## Conclusions

The fall in the prevalence of *D. immitis* in Australia since the introduction of MLs (monthly tablets and chews, top-spots and yearly depot injections) contrasts to the situation in most of the USA and Canada where the prevalence and extent of heartworm disease has increased despite the availability of these drugs [32–34]. In Australia, preventative drugs are widely available at veterinary clinics, pet stores, supermarkets and on the internet via online veterinary wholesalers, providing easy access to prophylactic therapy [13, 35]. More restricted availability of MLs in North America, reduced competition in the marketplace and possibly higher cost (as a result) may in part explain why heartworm disease has been virtually eradicated from NSW in Australia, in contrast to most of the USA [36]. Additionally, NSW has been in extended drought conditions for three years, which presumably has reduced the availability of mosquito vectors. In contrast to NSW, where we did not demonstrate canine heartworm infection in dog cohorts receiving no or intermittent prevention, Queensland pig-hunting dogs are identified as the at-risk population with 21% *D. immitis* antigen prevalence in Central Queensland.

## Data Availability

The data analyzed in this article are included within the article.

## Abbreviations

NDA: no detectable antigen
NSW: New South Wales
MLs: macrocyclic lactones
ELISA: enzyme-linked immunosorbent assay
Mff: microfilariae
